# Characterization of Mitochondrial Proteome and Function in Luminal A and Basal-like Breast Cancer Subtypes Reveals Alteration in Mitochondrial Dynamics and Bioenergetics Relevant to Their Diagnosis

**DOI:** 10.3390/biom12030379

**Published:** 2022-02-28

**Authors:** Ariadna Jazmín Ortega-Lozano, Leopoldo Gómez-Caudillo, Alfredo Briones-Herrera, Omar Emiliano Aparicio-Trejo, José Pedraza-Chaverri

**Affiliations:** 1 Department of Biology, Faculty of Chemistry, National Autonomous University of Mexico (UNAM), Mexico City 04510, Mexico; arijol-sk@hotmail.com (A.J.O.-L.); pologoca@gmail.com (L.G.-C.); bhalfredo@gmail.com (A.B.-H.); 2 Department of Cardio-Renal Physiopathology, National Institute of Cardiology “Ignacio Chávez”, Mexico City 14080, Mexico; emilianoaparicio91@gmail.com

**Keywords:** luminal A breast cancer, basal-like breast cancer, MCF7 cell line, MDA-MB-231 cell line, mitochondria dynamics, mitochondrial biogenesis, reactive oxygen species (ROS), mitochondrial proteome

## Abstract

Breast cancer (BC) is the most prevalent cancer and the one with the highest mortality among women worldwide. Although the molecular classification of BC has been a helpful tool for diagnosing and predicting the treatment of BC, developments are still being made to improve the diagnosis and find new therapeutic targets. Mitochondrial dysfunction is a crucial feature of cancer, which can be associated with cancer aggressiveness. Although the importance of mitochondrial dynamics in cancer is well recognized, its involvement in the mitochondrial function and bioenergetics context in BC molecular subtypes has been scantly explored. In this study, we combined mitochondrial function and bioenergetics experiments in MCF7 and MDA-MB-231 cell lines with statistical and bioinformatics analyses of the mitochondrial proteome of luminal A and basal-like tumors. We demonstrate that basal-like tumors exhibit a vicious cycle between mitochondrial fusion and fission; impaired but not completely inactive mitochondrial function; and the Warburg effect, associated with decreased oxidative phosphorylation (OXPHOS) complexes I and III. Together with the results obtained in the cell lines and the mitochondrial proteome analysis, two mitochondrial signatures were proposed: one signature reflecting alterations in mitochondrial functions and a second signature exclusively of OXPHOS, which allow us to distinguish between luminal A and basal-like tumors.

## 1. Introduction

Breast cancer (BC) is the neoplasia with the highest incidence and mortality among women worldwide. According to World Health Organization (WHO) data, 2.3 million women were diagnosed with BC and 685,000 deaths in the world in 2020, making it a global health problem [1]. BC is a disease characterized by a highly biological heterogeneity, which is noted by specific pathologic features, clinical behavior differences and different molecular alterations [2]. Estrogen receptor (ER), progesterone receptor (PR), and expression of the human epidermal growth factor receptor 2 (HER2) are classical immunohistochemistry markers that classify BC into four main molecular subtypes: luminal A, luminal B, HER2-enriched, and basal-like [3,4]. This molecular classification provides an accurate diagnosis of BC; in addition, it is also valuable for the prediction of tumor behavioral to chemotherapy. The luminal A subtype is the most common, comprising over 70% of all cases [5], and is ER and/or PR positive, HER2 negative, and proliferator marker Ki-67 <14%. Luminal A tumors are low-grade, they grow slowly, and they have the best prognosis, unlike the basal-like subtype, which comprises about 20% of all cases; this tumor type is biologically more aggressive and has the worst prognosis among all BC subtypes [4,6]. Basal-like tumors are also known as triple-negative, as they lack ER, PR, and HER2 expression; consequently, these tumors do not benefit from hormonal therapy or medicines that target HER2 protein receptors. Therefore, it is necessary to find a targeted therapy for basal-like patients [7].

Based on the above, we focus on the luminal A and basal-like subtypes in our present work. This is because the luminal A subtype, as mentioned, is the molecular subtype of BC with the highest incidence and the best prognosis compared to the three remaining subtypes. Although the basal-like subtype is the subtype with the lowest incidence, it is characterized by being the one with the worst prognosis and therapeutically abandoned since currently there are no biomarkers towards which to direct a specific treatment. In addition, these molecular subtypes of BC were chosen to find differences between them, and which could contribute to enriching the molecular classification of this disease.

Mitochondrial dysfunction is a crucial feature of cancer and is associated with aggressiveness [3]. The altered metabolism in cancer cells is a consequence of changes or defects in the mitochondrial bioenergetics [4]. In addition, there is evidence that cancer cells have altered their mitochondrial dynamics (the balance between mitochondrial fusion and fission), which is directly related to the change in mitochondrial biogenesis and turnover, giving proliferative and survival advantages for tumor cells [5,6].

Cancer cells are under metabolic changes; this metabolic alteration of mitochondria has a crucial role in tumorigenesis [3]. Currently, the mitochondrial alterations, regarding the dynamics and function, are still unknown among BC subtypes and their relationship with bioenergetics mitochondrial metabolism.

Despite studies reflecting the importance of mitochondrial dynamics in cancer and its relationship to bioenergetics and alterations in mitochondrial function, these alterations have been little studied in the molecular subtypes of BC [8,9].

This work aimed to find the differences in mitochondrial bioenergetics and function associated mainly with changes in mitochondrial dynamics between luminal A and basal-like subtypes. Comparing functional mitochondrial studies and evaluating mitochondrial dynamics in MCF7 and MDA-MB-231 cell lines of BC, representative of luminal A and basal-like subtypes, respectively [7], revealed that the MDA-MB-231 cell line was characterized by more remarkable mitochondrial alteration associated with changes in energy metabolism. Additionally, to further deepen the findings found in breast cancer cell lines, a mitochondrial proteome analysis was performed by statistical analysis of the proteome of BC biopsies classified as luminal A and basal-like from the experiments of Mertins et al. [10]. A mitochondrial proteomic signature showing differences in the expression of mitochondrial proteins involved in mitochondrial bioenergetics and mitochondrial dynamics processes was proposed for luminal A and basal-like subtypes of BC.

## 2. Materials and Methods

### 2.1. Cell Culture

The MCF7 cell line was provided by Dr. Alejandro García-Carrancá and MCF10A MDA-MB-231 cell lines were provided by Dr. Alejandro Zentella-Dehesa. The MCF7 and MDA-MB-231 BC cell lines were cultured in RPMI-1640 media supplemented with 10% fetal bovine serum (FBS) and 1% antibiotic-antimycotic. MCF10A breast epithelial cell was cultured in DMEM/F12 media supplemented with 10% FBS, 1% antibiotic-antimycotic, 0.5 µg/mL hydrocortisone, 10 ng/mL endothelial growth factor (EGF), 5 µg/mL insulin. Cells were cultured at 37 °C and 5% CO_2_ in a humidified atmosphere. The culture medium was renewed every third day until the cell cultures reached a confluence of 80–90%.

### 2.2. Western Blot

MCF7, MDA-MB-231 and MCF10A cells were washed twice with cold phosphate-buffered saline (PBS) and lysed in radioimmunoprecipitation assay (RIPA) buffer (40 mM Tris-HCl, 150 mM NaCl, 2 mM EDTA, 5 mM NaF, 1 mM Na_3_VO_4_, 1 mM PMSF, 1 mM Na_4_P_2_O_7_, 0.5% sodium deoxycholate, 0.2% SDS, pH 7.4 and 1× protease inhibitors cocktail) for 30 min at 4 °C with stirring. Lysates were centrifugated at 15,000× *g* for 10 min at 4 °C, and the supernatant was collected and stored at −70 °C until the experiment was carried out. Protein concentrations were quantified by Bradford assay. Equal amounts of protein (30 µg) were denatured by dilution on 6X Laemmli buffer (60 mM Tris-HCl pH 6.8, 2% SDS, 10% glycerol, 5% β-mercaptoethanol, 0.01% bromophenol blue) and immersion on boiling water for 5 min, except those employed to determine the subunit levels of mitochondrial complexes (CI-NDUFB8 and CIV-MTCO1). The samples were separated on 12% sodium dodecyl sulfate (SDS)–polyacrylamide gel electrophoresis (SDS/PAGE). Proteins were transferred onto Immobilion PVDF membranes for fluorescent application on wet transfer. Membranes were blocked for 1 h in at room temperature (RT) in Tris-buffered saline with 0.1% Tween-20 (TBST) containing 5% nonfat dry milk and incubated overnight at 4 °C with the appropriate primary antibodies (Table S1). Then, the membranes were incubated for 2 h at RT in darkness with the corresponding IRDye^®^ fluorescent secondary antibodies (1:10,000). Proteins bands were detected in an Odyssey Sa Infrared System (LI-COR Biosciences, Lincoln, NE, USA). Signals were then processed employing the software Image Studio™ 5.2 (LI-COR Biosciences). We used the blots stained with Ponceau red S as loading control due to the bioenergetics differences and structural changes in the cytoskeleton of the cell lines used in our study [11]. For this purpose, after the transfer, the membrane was incubated in 1% Ponceau S solution for 2 min. Immediately, this membrane was rinsed with phosphate-buffered saline (PBS) to remove staining saturation [12]. The membrane was then inserted between transparent sheets and scanned at 300 dpi as a JPG document using a standard scanner (HP Scanjet G4050). Densitometry data for total protein staining images with Ponceau S were obtained from all visible proteins in each complete lane. All data normalization processes were performed by dividing the target protein value by the value of the chosen loading control [13]. Densitometric analysis was performed using ImageJ software (National Institutes of Health, Bethesda, MD, USA, https://imagej.nih.gov/ij/index.html, accessed on 10 August 2021) on expression of fusion and fission markers: optic atrophy 1 (Opa1) (*n* = 9), mitofusin 1 (Mfn1) (*n* = 6), mitofusin 2 (Mfn2) (*n* = 6), and dynamin-1-like protein (Drp1) (*n* = 9); mitochondrial biogenesis markers: proliferator-activated receptor γ co-activator-1alpha (PGC-1α) (*n* = 6), and nuclear respiratory 2 (NRF2) (*n* = 3); mitophagy markers: PTEN-induced kinase 1 (PINK1) (*n* = 6), ubiquitin binding protein p62 (p62) (*n* = 3), Parkin RBR E3 ubiquitin-protein ligase (Parkin) (*n* = 3), and microtubule-associated proteins 1A/1B light chain 3B (LC3-II) (*n* = 3); oxidative metabolism marker: adenine monophosphate-activated protein kinase (AMPK) (*n* = 12); glycolytic metabolism marker: hypoxia-inducible factor 1α (HIF1α) (*n* = 12); and total oxidative phosphorylation rodent Western blot (WB) antibody cocktail (OXPHOS) (*n* = 3).

### 2.3. Mitochondrial Membrane Potential (ΔΨm) Assay

To measure ΔΨm by JC-1, 10,000 cells were cultured in a 96-well plate, and after 24 h, cells were incubated with dye JC-1 (1µg/mL) on non-supplemented medium at 37 °C for 30 min. The cells were washed twice with PBS and then replaced with fresh medium to remove the excess probe. Depolarized-related (green) fluorescence was measured at 525 nm, and the polarized-related (red) signal was read at 590 nm; both emissions were obtained at 488 nm excitation. Data and representative images were obtained a Cytation 5 Cell Imaging Reader (Biotek Instruments, Inc., Winoosky, VT, USA), with GFP and RFP filters. The JC-1 fluorescence was quantified as the red fluorescence/green fluorescence ratio in MCF10A (*n* = 7), MCF7 (*n* = 12), and MDA-MB-231 (*n* = 11) cell lines. The data were obtained from three biological replicates.

### 2.4. Estimation of Mitochondrial Mass

The protocol used to estimate of mitochondrial mass was based on the previous methodology from [14] with some modifications. Around 10,000 cells were seeded on 96-well black plates with the transparent bottom at the previously described culture conditions. After 24 h, the nuclei and mitochondria were stained with 1 μg/mL Hoechst and 500 nM MitoTracker Green^®^, on non-supplemented medium at 37 °C for 30 min. To remove the excess probe, cells were washed three times with PBS; fresh supplemented medium was placed on cultures. Hoechst fluorescence was measured at 360 nm excitation and 460 nm emission, while MitoTracker green fluorescence was measured at 480 nm excitation and 521 nm emission. Representative images were taken by using a Cytation 5 with the GFP and the DAPI filters while using Gen5™ 3.0 software (Biotek) for data acquisition and analysis. Mitochondrial mass levels was quantified as MitoTracker green fluorescence intensity/cell ratio in MCF10A, MCF7, and MDA-MB-231 cells. The number of cells was measured with ImageJ software (National Institutes of Health, Bethesda, MD, USA, https://imagej.nih.gov/ij/index.htm, accessed on 10 August 2021). The data were obtained from three biological replicates.

### 2.5. Determination of Mitochondrial ROS Production

Mitochondrial ROS production was measured using the fluorescent probe, Mito-SOX™ Red was based on the methodology previously from [15] with some modification. Around 10,000 cells were seeded on 96 well-black plates with a transparent bottom at the previously described culture conditions. After 24 h, the cells were incubated with 1 μg/mL Hoechst and 5 µM MitoSOX on non-supplemented medium at 37 °C for 30 min. To remove the excess probe, cells were washed three times with PBS; fresh supplemented medium was placed on cultures. Hoechst fluorescence was measured at 360 nm excitation and 460 nm emission, while MitoSOX red was measured at 510 nm excitation and 580 nm emission. ROS production was quantified as the MitoSOX red/cell ratio in MCF10A, MCF7, and MDA-MB-231 cells. Representative images were taken by using a Cytation 5 while using Gen5™ 3.0 software for data acquisition and analysis. The number of cells was measured with ImageJ software (National Institutes of Health, Bethesda, MD, USA, https://imagej.nih.gov/ij/index.htm, accessed on 10 August 2021). The data were obtained from three biological replicates.

### 2.6. Cell Respirometry

The oxygen consumption experiments in cells were evaluated with a high-resolution respirometry equipment O2K (Oroboros Instruments, Innsbruck, Austria) according to the previous protocol from [16] with some modification. Cells were washed with PBS, harvested with trypsin, and quantified by trypan blue assay. Determinations were made with approximately 1 million cells in 2 mL of culture medium with 10% FBS at 37 °C. The respiratory parameters evaluated were: (1) Routine respiration, corresponding to oxygen consumption of the cells; (2) Leak of the respiration, corresponding to oxygen consumption in the presence of 5 µM oligomycin; (3) Respiratory control (RC), that corresponding to the routine/leak ratio; (4) Respiration attributable to oxidative phosphorylation (P), was calculated by the difference between Routine and Leak. All parameters were corrected by subtracting the non-mitochondrial respiration, obtained by the addition of 1 µM rotenone, 5 µM antimycin A, 100 µM sodium azide and normalized by the number of cells determined by trypan blue.

### 2.7. Statistical Analysis

The results obtained from the cell lines were analyzed with the R package Rapport [17] to eliminate outliers. The data were tested for normality and analyzed by one-way analysis of variance (ANOVA), followed by the Tukey’s multiple comparisons test. Data were plotted to show three or more independent experiments, and every figure shows the mean with standard deviation. All data were analyzed using the software R (version 4.1.0, Foundation for Statistical Computing).

### 2.8. Analysis of the Mitochondrial Proteome in Breast Cancer Tumors

To complement and validate the changes found in mitochondrial dynamics and mitochondrial bioenergetics between cell lines of BC, global proteome abundance data from 43 tumors (18 classified as basal-like, 22 luminal A, and 3 non-cancerous as controls) stored in supplementary Table S3 reported by Mertins et al. [10] were selected to be reanalyzed. Briefly, in order to correlate the genome, transcriptome and proteome in luminal A, luminal B, HER2, and basal-like molecular subtypes of BC, Mertins et al. [10] analyzed using tandem mass spectrometry (MS) the total proteome of 102 cancerous tumors histopathological and molecularly classified by The Cancer Genome Atlas (TCGA), in the four main intrinsic subtypes and three controls. A detailed explanation of the experiment and spectrometry analysis of tumors can be seen in [10].

In the first part of our analysis, all the proteins identified and quantified by Mertins et al. [10] were cross-referenced with a list of mitochondrial or mitochondrial traffic proteins reported by MitoMiner [18]. Of these mitochondrial proteins, only those with abundance values in at least 50% of the samples were selected.

Next, an exploratory analysis of the abundance data was carried out with the Rap-port of R package [17] to eliminate from the analysis, both the samples as proteins with extreme behavior.

After eliminating the extreme data, a hierarchical cluster analysis of the tumors by the Ward method based on the Euclidian distance matrix of the samples was carried out to find homogenous groups between subtypes. Fifteen tumor samples were eliminated because they were confounded in the same cancerous subtype of BC.

With 28 final samples (14 basal-like, 11 luminal A and 3 controls), missing values were imputed with the Random Forest method (missForest, R package) [19]. Next, a PCA was applied on the protein abundance correlation matrix [20] to obtain an abundance landscape of mitochondrial proteins for the subtypes and control group [21].

Finally, only proteins with an absolute value of association equal or greater than 0.5 with both first components [22] were selected for comparative overrepresentation analysis based on Gene Ontology [23]. Overrepresentation was performed online employing the Gene List Analysis tool on the PANTHER Classification System site. As inputs, we up-loaded the official gene symbols as identifiers. We select those involved in the dynamics processes, mitochondrial biogenesis, mitophagy, mitochondrial ROS, mitochondrial membrane potential and mitochondrial metabolism to conform to the mitochondrial signature of luminal A and basal-like subtypes. A flow chart of the processing and analysis of these data can be seen in Figure S1.

## 3. Results

### 3.1. Reduction in Drp1-Related Mitochondrial Fission in the Basal-like Cell Line

**Figure 1:**
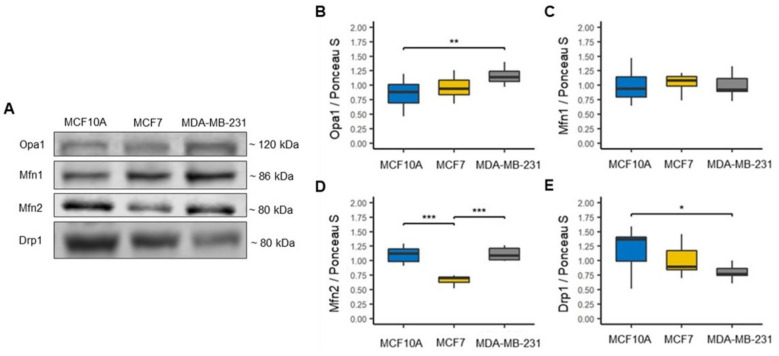
Mitochondrial dynamics in breast cancer cell lines. (**A**) Representative blots. (**B**–**D**) Expression of fusion markers, optic atrophy 1 (Opa1), *n* = 9; mitofusin 1 (Mfn1), *n* = 6; mitofusin 2 (Mfn2), *n* = 6. (**E**) Expression of fission marker, dynamin-1-like protein (Drp1), *n* = 9. Densitometry values were normalized by Ponceau S red staining. The data are presented as mean ± SD. * *p* < 0.05, ** *p* < 0.01, *** *p* < 0.001.

Alteration in the mitochondrial dynamics in cancer cells are tightly associated with mitochondrial morphology, with alterations in mitochondrial mass, mitochondrial biogenesis, dysregulation of the bioenergetics and redox functions [24,25]. We first assessed the expression of the fusion markers mitofusins 1 and 2 (Mfn1 and Mfn2) and optic atrophy type 1 (Opa1), as well as expression of the fission marker dynamin-related protein 1 (Drp1) in cell lines MCF7 representative luminal A subtype of BC, MDA-MB-231 representative basal-like subtype of BC, and MCF10A as a non-tumorigenic (control) (Figure 1A) [7]. We found a significant increase in Opa1 in the MDA-MB-231 cell line compared to the MCF10A cell line; however, no significant differences were found between the BC cell lines, but a tendency to increase this protein can be observed in the cell line MDA-MB-231 compared to the MCF7 cell line (Figure 1B). In the Mfn1 protein, no expression changes were found between cell lines (Figure 1C). However, in the Mfn2 protein, a significant decrease was found in the MCF7 cell line compared to the MCF10A cell line and a significantly higher expression in the MDA-MB-231 cell line compared to MCF7 cell line (Figure 1D). In addition, we observed that Drp1 protein expression decreased in both cell lines of BC compared to the MCF10A cell line, although this decrease was only significant in the MDA-MB-231 cell line (Figure 1E). To our surprise, it was found that the decrease in Drp1 protein expression was mainly marked in the MDA-MB-231 cell line that is representative of the basal-like subtype. In contrast, previous studies have reported that mitochondrial fission tends to increase in cancer cells and that this increase can be associated with the aggressiveness of this disease [26]. These data suggest changes in mitochondrial control and quality and important differences in mitochondrial biology among BC subtypes and that mitochondrial fission in basal-like BC may be mediated by other Drp1-independent proteins.

### 3.2. Mitochondrial Biogenesis and Mitophagy in MCF7 and MDA-MB-231 Cells

**Figure 2:**
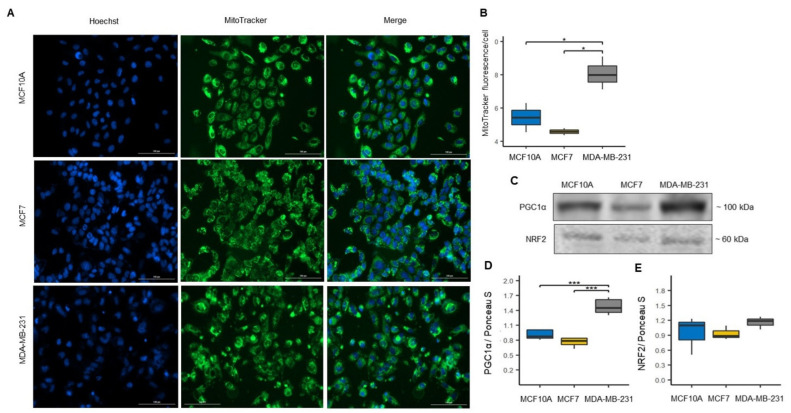
Increased mitochondrial mass in MDA-MB-231 cell line. (**A**) Confocal microscopy images of MitoTracker Green fluorescence to assess mitochondrial mass in MCF10A, MCF7 and MDA-MB-231 cells, and total nuclei were stained with Hoechst (blue). (**B**) Mitochondrial mass levels were quantified as MitoTracker green fluorescence intensity/cell ratio. Data were obtained from three biological replicates. The data are presented as mean ± SD of cells (*n* = 151–251). * *p* < 0.05. (**C**) Representative blots. (**D**,**E**) Expression of mitochondrial biogenesis markers: peroxisome proliferator-activated receptor-gamma coactivator-1alpha (PGC-1α), *n* = 6 and nuclear respiratory factor 2 (NRF2), *n* = 3. Densitometry values were normalized by Ponceau S red staining. The data are presented as mean ± SD. *** *p* < 0.001.

Mitochondrial homeostasis is preserved by coordination between mitochondrial biogenesis and mitophagy [27]. To assess the effect of differences in mitochondrial dynamics on mitochondrial mass and biogenesis among BC cell lines, we measured mitochondrial mass using MitoTracker Green probe (Figure 2A). We found a significant increase in the fluorescence intensity of the MitoTracker Green in the MDA-MB-231 cell line compared to in the control and MCF7 cell lines, suggesting a higher mitochondrial mass content in the cell line representative of basal-like molecular subtype of BC. In contrast, the MitoTracker Green intensity values obtained in the MCF7 cell line suggest a lower mitochondrial mass content than the control and MDA-MB-231 cell lines (Figure 2B). To validate the mitochondrial mass results found in the cell lines of BC, we evaluated the expression of proliferator-activated receptor γ co-activator-1alpha (PGC1α) and nuclear respiratory 2 (NRF2) proteins involved in mitochondrial biogenesis (Figure 2C) [27]. We found a correlation with the data obtained by MitoTracker Green; we found a significant increase in the expression of the PGC1α protein in the MDA-MB-231 cell line compared to the MCF10A and MCF7 cell lines (Figure 2D). Although we found no significant changes in NRF2 protein expression, we observed an increment trend in the expression of this protein in the MDA-MB-231 cell line (Figure 2E).

**Figure 3:**
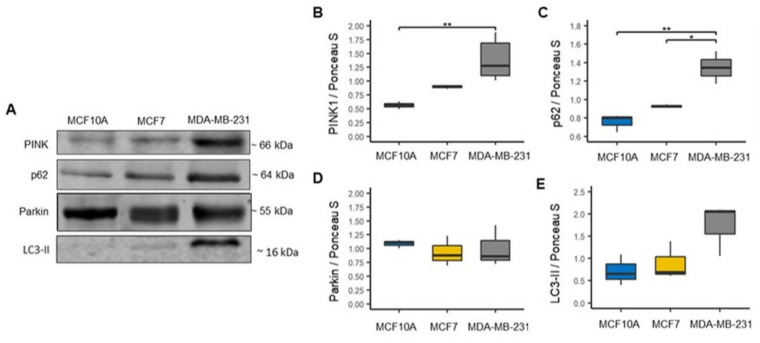
Increased mitophagy markers in the MDA-MB-231 cell line. (**A**) Representative blots. (**B**–**E**) Expression of mitophagy markers, PTEN-induced kinase 1 (PINK1), *n* = 6; ubiquitin-binding protein p62 (p62), *n* = 3; Parkin, *n* = 3; and microtubule-associated proteins 1A/1B light chain 3B (LC3-II), *n* = 3. Densitometry values were normalized by Ponceau S red staining. The data are presented as mean ± SD. * *p* < 0.05, ** *p* < 0.01.

In addition, we evaluated mitophagy markers since it is a mitochondrial quality control process and a mechanism of mitochondrial mass regulation by which dysfunctional mitochondria are eliminated [28,29]. Thus, we evaluated the expression of mitophagy-related proteins PTEN-induced kinase 1 (PINK1) and Parkin, and autophagic proteins, ubiquitin-binding protein p62 (p62) and microtubule-associated proteins 1A/1B light chain 3B (LC3-II) (Figure 3A). We found that PINK1 and p62 showed a significant increase in the MDA-MB-231 cell line compared to the MCF10A cell line (Figure 3B,C). Additionally, we found a significant increase in the expression of p62 in the MDA-MB-231 cell line compared to the MCF7 cell line (Figure 3C). However, we did not find significant changes in Parkin LC3-II proteins expression between the cell lines (Figure 3D,E). Although we found no significance changes in LC3-II, we observed an increment trend in the expression of this protein in the MDA-MB-231 cell line (Figure 3E). Overall, the results of both mitochondrial biogenesis and mitophagy correlate with the previously obtained results with the mitochondrial dynamics markers. This is because increased mitochondrial fusion processes are related to increased mitochondrial mass as observed in the MDA-MB-231 line [30].

Additionally, these results suggest alterations in mitochondrial quality control and reveal differences in mitochondrial biology between luminal A and basal-like BC subtypes.

### 3.3. Mitochondrial Uncoupling in Luminal A and Basal-like Cell Lines of Breast Cancer

**Figure 4:**
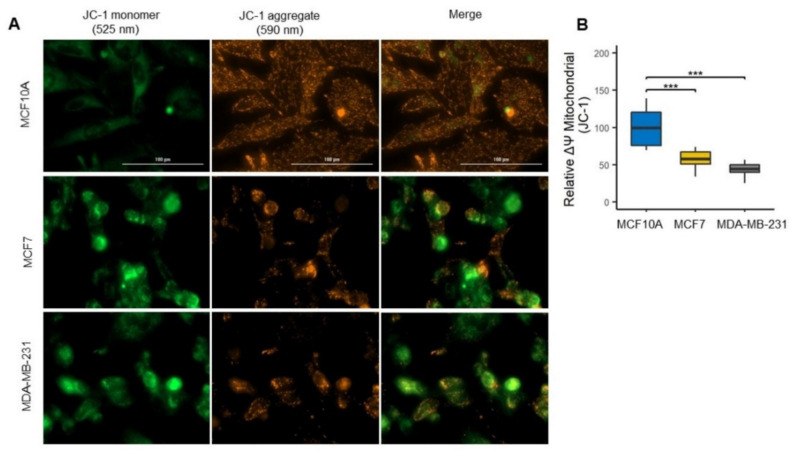
Decrease in mitochondrial membrane potential (ΔΨm) in the MDA-MB-231 cell line. (**A**) ΔΨm levels were assessed by JC-1 probe; MCF10A cells with highly polarized mitochondria accumulate JC-1 dye in the mitochondrial matrix, forming bright red fluorescent J aggregates. Increased green fluorescence indicates decreased mitochondrial potential. (**B**) The JC-1 fluorescence was quantified as the red fluorescence/green fluorescence ratio in MCF10A (*n* = 7), MCF7 (*n* = 12), and MDA-MB-231 (*n* = 11) cell lines. Data were obtained from three biological replicates. The data were normalized with the values of MCF10A cells and are presented as mean ± SD. *** *p* < 0.001.

To assess this functional state and investigate the ATP-generating capacity of mitochondria, we evaluated the membrane potential as a driver for ATP generation (Figure 4A) [31]. As expected, the ΔΨm decreased in both BC cell lines (Figure 4A,B); however, although no significant differences were found between the BC cell lines, it is observed that the ΔΨm is little higher in the MCF7 cell line than MDA-MB-231 cell line, as indicated by a higher ratio of JC-1 red to green fluorescence (Figure 4B). These results correlate with the results found in mitophagy, suggesting that reduction in ΔΨm favors mitochondrial mass reduction by mitophagy induction and that mitochondrial inner membrane is better maintained in the luminal A MCF7 cell line than in basal type MDA-MB-231 cell line.

### 3.4. Overexpression of HIF-1α Associated with Metabolic Reprogramming in MCF7 and MDA-MB-231 Cell Lines

**Figure 5:**
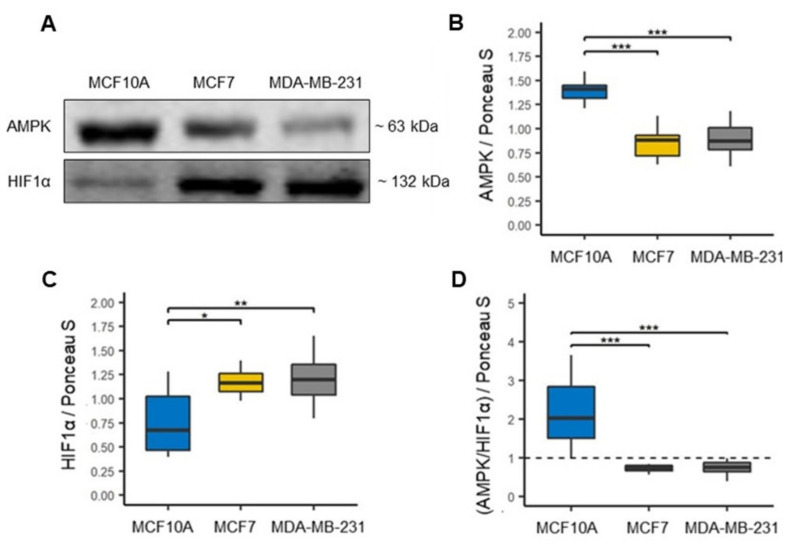
Metabolic reprogramming in breast cancer cell lines. (**A**) Representative blots. (**B**) Expression of oxidative metabolism marker, adenine mono-phosphate-activated protein kinase (AMPK), *n* = 12. (**C**) Expression of glycolytic metabolism marker, hypoxia-inducible factor 1α (HIF1α), *n* = 12. (**D**) AMPK/HIF1α ratio in MCF10A, MCF7 and MDA-MB-231 cell lines, a ratio less than one (black line) reflects a preferential glycolytic metabolism. Densitometry values were normalized by Ponceau S red staining. The data are presented as mean ± SD. * *p* < 0.05, ** *p* < 0.01, *** *p* < 0.001.

Although glycolysis is a dominant metabolism in cancer cells, there is evidence that mitochondrial OXPHOS is also used by cancer cells [32,33,34]. Moreover, since the BC cell lines studied here showed a different mitochondrial function, we analyzed the expression of adenine monophosphate-activated protein kinase (AMPK) and hypoxia-inducible factor 1α (HIF1α) (Figure 5A) [35], to evaluate the relative contribution of oxidative and glycolytic metabolism, respectively, in both BC cell lines. We first observed a significant decrease in AMPK expression in both cell lines of BC compared to the MCF10A cell line, suggesting that both MCF7 and MDA-MB-231 cell lines decreased oxidative metabolism (Figure 5B). In contrast, HIF1α expression was found to be significantly increased in both cell lines of BC compared to the MCF10A cell line (Figure 5C), suggesting that both MCF7 and MDA-MB-231 lines had a preference for glycolytic metabolism. Then, through the AMPK/HIF1α ratio, we found that it was less than one in the two cell lines of BC (Figure 5D), which reaffirms that both cell lines have a predominantly glycolytic metabolism compared to the cell line MCF10A.

### 3.5. Functional Status Mitochondrial in Luminal A and Basal-like Breast Cancer Cell Lines

**Figure 6:**
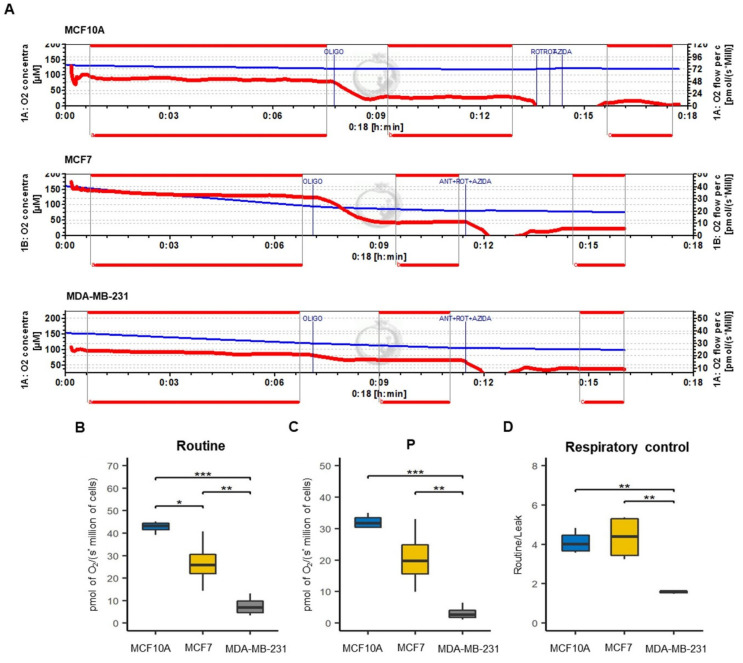
Mitochondrial respiratory efficiency of breast cancer cell lines. (**A**) Schematic representation of O_2_ consumption rate by addition in order of OLIGO (oligomycin) and ANT + ROT + AZIDA (antimycin A plus rotenone plus sodium azide) inhibitors to determine the respiratory parameters in MCF10A, MCF7 and MDA-MB-231 cell lines. The blue line shows the oxygen concentration in the chamber while the red line indicates the oxygen consumption rate. (**B**) Cellular routine respiration (Routine), that correspond to oxygen consumption of the cells. (**C**) Respiration associated with oxidative phosphorylation (P); (**D**) respiratory control (RC) calculated as routine/leak ratio. All parameters were corrected by subtracting the non-mitochondrial respiration, obtained by the addition of ANT + ROT + AZIDA. The data are presented as mean ± SD, *n* = 3–4. * *p* < 0.05, ** *p* < 0.01, *** *p* < 0.001.

To assess the compromised function of mitochondria and to confirm the finding of decreased OXPHOS in BC cell lines, we evaluated cellular oxygen consumption (Routine) (Figure 6A), respiration associated with oxidative phosphorylation (P) (Figure 6B), and mitochondrial respiratory efficiency (RC) (Figure 6C). We found a significant decrease in Routine and P parameters in BC cell lines, notably more marked for the MDA-MB-231 than for MCF7. These confirm the reduction in mitochondria bioenergetics, specifically in OXPHOS, in cell lines of BC, which agrees with mitochondrial mass reduction. Furthermore, these results also suggest a higher mitochondrial decoupling in the MDA-MB-231 cell line, as is shown by a low RC value.

### 3.6. Increased ROS Production in the Basal-like Cell Line

**Figure 7:**
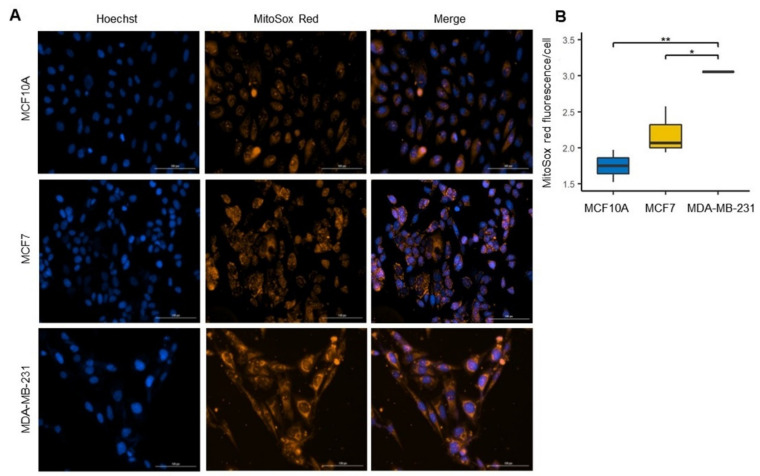
Increased levels of reactive oxygen species (ROS) in the MDA-MB-231 line. (**A**) Confocal microscopy images of mitochondrial ROS were stained by MitoSOX red (red) in MCF10A, MCF7 and MDA-MB-231 cell and total nuclei were stained with Hoechst (blue). (**B**) ROS production was quantified as MitoSOX red fluorescence intensity/cell ratio. Data were obtained from three biological replicates. The data are presented as mean ± SD of cells (*n* = 153–200). * *p* < 0.05, ** *p* < 0.01.

Increased ROS production is a feature associated with mitochondrial dysfunction [36,37]. Additionally, evidence associated increased ROS production with cancer cell malignancy [38,39]. MitoSOX red has been used to measure mitochondrial ROS production since it preferentially accumulates within the mitochondrial matrix [38,39,40]. To evaluate the differences in ROS generation, we used the MitoSOX red probe in cell lines of BC and control cell line (Figure 7A). We detected a significant increase in ROS in both cell lines of BC compared to the control cell line. Furthermore, we found that ROS production was higher in the MDA-MB-231 cell line than in the MCF7 cell line (Figure 7B); this strongly agreed with the mitochondrial uncoupling observed in this cell line. These results indicate a more remarkable mitochondrial alteration in the basal-like cell line, which may be related to alterations in the mitochondrial electron transport chain.

### 3.7. Landscape of the Mitochondrial Proteome of Luminal A and Basal-like Subtypes of Breast Cancer

After cross-checking the 12,553 proteins identified by Mertins et al. [10] against the Mitominer database [18], we were left with 1152 mitochondrial or mitochondrial-transiting proteins (Table S2). Of these, 51 proteins were eliminated because they had no abundance values in at least 50% of the samples or showed atypical abundance values. Nineteen samples were also eliminated from the analysis, two showing atypical abundance patterns and 17 confounded in the hierarchical cluster analysis (Figure S2). Finally, the analysis was carried out with 1103 proteins, 42 exclusively expressed in cancer (Table S3) and 1061 with abundance values in at least 50% of all samples (Table S4) and 28 samples.

**Figure 8:**
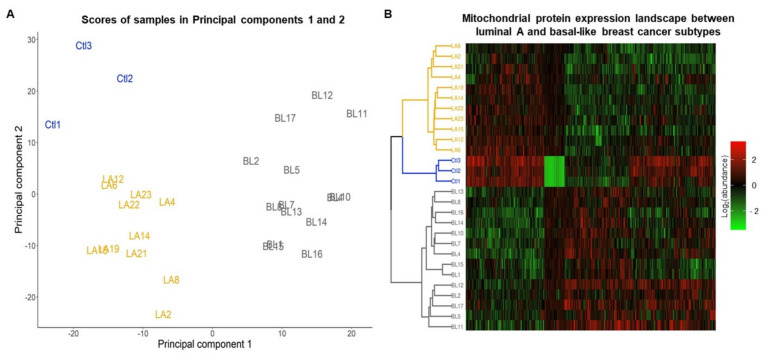
Analysis of the mitochondrial proteome of luminal A and basal-like tumors. (**A**) Principal component analysis of the mitochondrial proteome in samples control (Ctl), luminal A (LA), and basal-like (BL) breast cancer tumors. (**B**) Heatmap and hierarchical cluster analysis of mitochondrial proteins abundance identify in samples control, luminal A, and basal-like tumors of breast cancer.

The principal component analysis (PCA) showed that total protein abundance variation between samples is explained by 27 Principal Components (PCs); the first two PCs embrace 31% of whole abundance variability (Table S5) and can differentiate between luminal A and basal-like subtypes of BC and the control group (Figure 8A).

The heatmap shows that 477 proteins whose correlation with PC 1 or PC 2 is less than −0.5 or greater than 0.5 (Table S6) present a different abundance pattern between samples belonging to each of the subtypes and the control group (Figure 8B). Four patterns of expression can be distinguished in the heat map one in which a set of proteins are overregulated in luminal A and control samples and downregulated in basal-like, a second pattern in which proteins are downregulated in controls and moderately expressed in BC molecular subtypes, in the third pattern a set of proteins are downregulated in luminal A tumors and control and overexpressed in basal tumors and in the fourth pattern a set of proteins are observed with heterogeneous expression among molecular subtypes and control samples.

Additionally, a hierarchical clustering analysis applied to the abundance data in the heatmap revealed the formation of three groups. The first cluster (yellow lines) was enriched by the luminal A tumors, the control samples enriched the second cluster (blue lines), and the third cluster (gray line) was enriched by basal-like tumors, which reflected the differences in mitochondrial proteome abundance patterns between these BC subtypes (Figure 8B).

### 3.8. Signature Mitochondrial Alterations in Luminal Type A and Basal-like Breast Cancer

To validate and deepen the results found in cell lines on the mitochondrial processes of fusion (FUS), fission (FIS), mitophagy (MIT), ROS, ΔΨm (PMM) and mitochondrial membrane organization (MMO) processes, an overrepresentation analysis of these processes were performed using the proteins that presented an absolute value of association with the first two components equal to greater than 0.5, to distinguish between molecular subtypes. When the analysis of the overrepresentation of biological processes with mitochondrial proteins was performed, the process of mitochondrial biogenesis was not localized, possibly because mitochondrial biogenesis is a process that requires the intervention of the cell nucleus. To provide information on mitochondrial biogenesis, we chose the MMO process. Of the 519 mitochondrial proteins used for overrepresentation analysis (Table S7), 15,717 biological processes were found to be overrepresented (Table S8), and a total of 54 proteins were found to be involved in the biological processes analyzed in this study.

**Figure 9:**
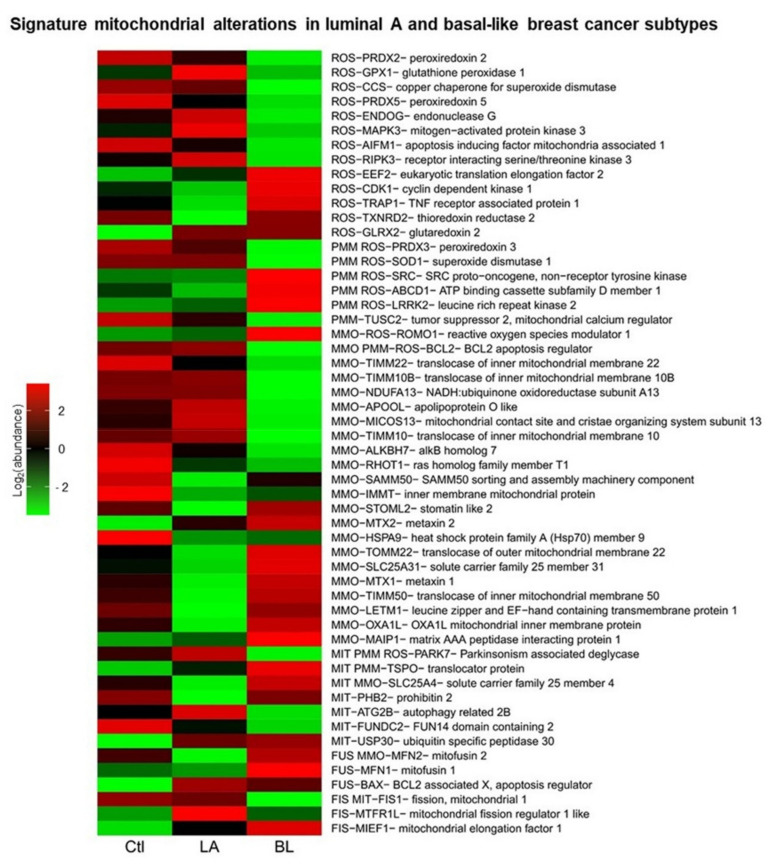
Signature mitochondrial alterations in luminal type A and basal-like breast cancer. Expression profiles of proteins involved in regulation and formation of reactive oxygen species (ROS), mitochondrial membrane potential (MMP), mitochondrial membrane organization (MMO), mitophagy (MIT), mitochondrial fusion (FUS) and mitochondrial fission (FIS), in control samples, luminal A and basal-like breast cancer tumors.

A mitochondrial signature was obtained with the 54 mitochondrial proteins that show us patterns of abundance of proteins involved in mitochondrial dynamics, biogenesis, mitophagy, potential and ROS (Figure 9). In the signature proposed here, it is observed that proteins may be involved in one or several processes.

Additionally, characteristic protein expression patterns were found for each of the samples. In luminal A tumors, we found overexpression of GPX1, CCS, ENDOG, MAPK3, and RIPK3 proteins involved in ROS; PRDX3 and SOD1 in the ROS and PMM processes; TIMM10B, NDUFA13, APOOL, MICOS13 and TIMM10 of the MMO process; BCL2 involved in the MMO, PMM and ROS processes; PARK7 in MIT, PMM and ROS processes; ATG2B in MIT; FIS1 in FIS and MIT and MTFR1L in FIS compared to basal-like BC tumors. However, we found underexpression of the proteins CDK1, TRAP1 and TXNRD2 related to ROS; SRC, ABCD1 and LRRK2 in PMM and ROS; ROMO1 in MMO and ROS; STOML2, TOMM22, SLC25A31, MTX1, TIMM50, LETM1, OXA1L and MAIP1 in MMO; TSPO, SLC25A4 and PHB2 in MIT; and MFN2 and MFN1 in FUS compared to basal-like BC tumors.

In basal-like tumors, we found the overexpression of EEF2, CDK1, TRAP1 and TXNRD2 involved in ROS; SRC, ABCD1 and LRRK2 in ROS and PMM processes; ROMO1 in ROS and MMO; STOML2, MTX2, TOMM22, SLC25A31, MTX1, TIMM50, LETM1, OXA1L and MAIP1 in MMO; PHB2 in MIT; TSPO in MIT and PMM; SLC25A4 in MIT and MMO processes; MFN1 in FUS; MFN2 in FUS and MMO processes; and MIEF1 in FIS compared to luminal A tumors. However, we found underexpression of the proteins: PRDX2, GPX1, CCS, PRDX5, ENDOG, MAPK3, AIFM1 and RIPK3 involved in ROS; TUSC2 in PMM; PRDX3 and SOD1 in PMM and ROS processes; TIMM22, TIMM10B, NDUFA13, APOOL, MICOS13, TIMM10, ALKBH7, RHOT1 in MMO; BCL2 in MMO, PMM and ROS processes; PARK7 in MIT, PMM and ROS processes; ATG2B and FUNDC2 in MIT; MTFR1L in FIS; and FIS1 in FIS and MIT processes compared to luminal A tumors.

Moreover, in the case of control samples, we found overexpression of the proteins: PRDX2, PRDX5 and AIFM1 involved in ROS; TUSC2 in PMM; ALKBH7, RHOT1, SAMM50, IMMT and HSPA9 in the MMO; and FUNDC2 in MIT compared to luminal A and basal-like breast cancer tumors. However, we found underexpression of EEF2 and GLRX2 proteins involved in ROS; MTX2 in MMO; USP30 in MIT; TSPO in MIT and PPM processes; BAX in FUS; and MTFR1L and MIEF1 in FIS compared to luminal A and basal-like breast cancer tumors.

In general, these previously described protein expression profiles allow us to distinguish between luminal A and basal-like tumors and distinguish between women with and without cancer. It is also worth mentioning that these results obtained in women with luminal A and basal-like BC correlate with the data found in MCF7 and MDA-MB-231 cell lines.

### 3.9. Protein Signature of OXPHOS in Luminal A and Basal-like Breast Cancer

In addition, we generated a signature that exclusively shows abundance patterns of OXPHOS proteins between BC molecular subtypes and control samples to delve deeper into mitochondria’s functional status and the potential it may have in detecting therapeutic targets of OXPHOS in BC [41,42].

**Figure 10:**
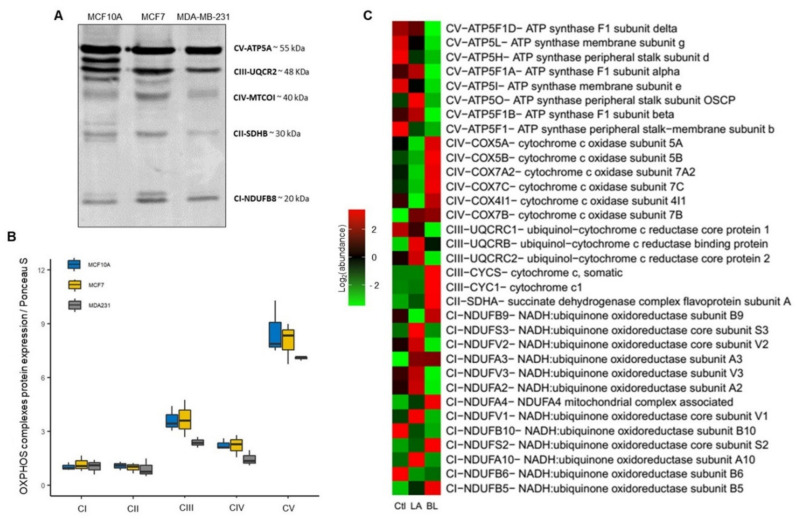
OXPHOS protein expression profiles in luminal A and basal-like subtypes of breast cancer. (**A**,**B**) Western blotting and expression of total oxidative phosphorylation (OXPHOS) cocktail in MCF10A, MCF7 and MDA-MB-231 cell lines. Densitometry values were normalized by Ponceau red staining. The data are presented as mean ± SD, *n* = 3. (**C**) Proteins expression profiles of OXPHOS complexes in control samples, luminal A, basal-like breast cancer tumors.

We first evaluated the expression of mitochondrial complexes in cell lines by WB, using a cocktail containing antibodies against a labile subunit when its complex is not assembled. The following subunits of the OXPHOS complexes were immunodetected: NADH: Subunit B8 of ubiquinone oxidoreductase (CI-NDUFB8), subunit B of the iron-sulfur succinate dehydrogenase complex (CII-SDHB), ubiquinol-cytochrome c reductase core protein 2 (CIII-UQCRC2), mitochondrial cytochrome c oxidase catalytic subunit (CIV-MTCO1) and ATP synthase F alpha subunit (CV-ATP5A) (Figure 10A). However, we found no significant differences, but we observed a tendency to decrease the expression of the CIII-UQCR2, CIV-MTCOI and the CV-ATP5A in the MDA-MB-231 line (Figure 10B).

In addition, we performed an overrepresentation analysis of OXPHOS with PCA proteins to generate expression profiles with OXPHOS proteins (Figure 10C).

We found that in luminal A tumors overexpression of the NDUFS3, NDUFV2, NDUFV3, NDUFA2, NDUFV1 and NDUFA10, and underexpression of the NDUFB9, NDUFA4, NDUFB10, NDUFS2 and NDUFB5 proteins of the CI; and underexpression of the COX5A, COX5B, COX7A2, COX7C and COX4I1 proteins of the CIV.

In basal-like tumors, we found overexpression of the NDUFB9, NDUFA4, NDUFS2 and NDUFB5 proteins of the CI; overexpression of the SDHA protein of the CII; overexpression of the CYCS and CYC1 proteins of the CIII and overexpression of the UQCR2 protein of the CIII, confirming the findings in cell lines by Western blot. Furthermore, in this same tumor type, we observed overexpression of the COX5A, COX5B, COX7A2, COX7C and COX4I1 proteins of the CIV; and underexpression of the ATP5F1D, ATP5L, ATP5H, ATP5F1A, ATP5I, ATP5O, ATP5F1B and ATP5F1 proteins of the CV.

We also observed heterogeneous expression of CI and CIII proteins in the two molecular subtypes of BC.

These findings show that the defects in the OXPHOS complexes impaired mitochondrial function, as found in past experiments. We believe that these marked differences in expression between the proteins of the OXPHOS complexes could be of diagnostic value, as they show a correlation with the aggressiveness of the type of BC.

In brief, analysis of mitochondrial protein expression shows that through the mitochondrial proteome it is possible to distinguish between BC samples and non-cancerous and to identify molecular subtypes. In addition, the analysis captures bioenergetics differences between BC types but also captures mitochondrial functional differences and their association with the aggressiveness that characterizes each type of BC. These results highlight the importance of studying differences in mitochondrial function between BC subtypes.

## 4. Discussion

Despite all the evidence of the critical role of mitochondria in cancer, few studies to date have focused on showing mitochondrial differences between BC subtypes, as well as few that reflect alterations in mitochondrial dynamics and their relationship to different mitochondrial and cellular processes.

The balance between mitochondrial fusion and fission is crucial to the preservation of mitochondrial function. Changes in mitochondrial dynamics have been associated with alteration in mitochondrial content, bioenergetics function and malignancy in cancer cells [43,44].

In different types of cancer, including BC, it has been reported that increased mitochondrial fission is associated mainly with Drp1 and Fis1 proteins and decreased mitochondrial fusion, which has been associated with poor prognosis and cancer aggressiveness [45,46,47,48]. This work demonstrates differential expression of proteins related to mitochondrial dynamics between luminal A and basal-like of BC. From both analyses of BC cell lines and analysis of the mitochondrial proteome in women with BC, we found decreased expression of the mitochondrial fission proteins Drp1 in MDA-MB-231 cell line (Figure 1E) and Fis1 in basal-like tumors (Figure 9). In contrast, Mfn 1 and 2 proteins overexpression was observed in basal-like tumors (Figure 9) and overexpression of Opa1 in the MDA-MB-231 cell line (Figure 1B). These findings suggest an increase in mitochondrial fusion for basal-like tumors. However, proteomics data suggest a decrease in mitochondrial fusion in luminal A subtype tumors. Similar results have been reported in gastric and lung cancer, in which Mfn2 overexpression has also been found [49,50]. Additionally, in Y. Lou et al.’s study, they also observed that silencing of Mfn2 in lung cancer results in inhibition of cell proliferation without impacting cell apoptosis [50]. Likewise, a recent study in patients with BC provided evidence that mitochondrial fission inhibits metastasis in triple-negative BC and that genes associated with mitochondrial fission correlate with better survival. In comparison, fusion genes correlate with worse survival [26]. In addition, these findings suggest that the mitochondrial dynamics have a more complex effect on BC cells than has been considered ultimately, which has not been fully elucidated.

Moreover, according to the mitochondrial proteome analysis (Figure 9), we believe that mitochondrial elongation factor 1 (MIEF1) may be a central regulator of mitochondrial fission in basal-like tumors. Research in colorectal cancer has reported that mitochondrial division is affected by MIEF1 [51]. However, few studies have explored the influence of MIEF in BC.

On the other hand, this controversial finding may be related to the increased mitochondrial mass observed in basal-like cells (Figure 2A,B). It is well established that both mitochondrial biogenesis and mitophagy are essential to preserving mitochondrial quality control [52,53]. As mentioned, we surprisingly found an increase in PGC1α biogenesis-related protein in the MDA-MB-231 cell line (basal-like) when compared to MCF7 (luminal A) and MCF10A (control) cell lines (Figure 2D). This increase in PGC1α levels may be the result of a compensatory mechanism induced by the decrease in mitochondrial respiration and decoupling in OXPHOS (Figure 6B,C), as PGC-1α-mediated mitochondrial biogenesis increases the number of mitochondria and replaces damaged mitochondria. Furthermore, mitofusin-mediated fusion decreases the damage that accumulates in mitochondria [30]. Therefore, these data suggest a significantly altered mitochondrial function in MDA-MB-231 BC than in MCF7. Furthermore, we found a significant increase in the expression of PINK1 and p62 mitophagy proteins that contribute to removing mitochondria damage (Figure 3B,C). Additionally, although no significant difference in LC3-II expression was observed among the BC cell lines, an increasing trend was observed, especially in the MDA-MB-231 cell line (Figure 3E). This increase in the expression of mitophagy-associated proteins may be related to the decrease in both OXPHOS in both cell lines of BC. In addition to decreased mitochondrial function, we show that activation of mitophagy is a response to the decreased mitochondrial membrane potential (Figure 4A,B). Consistent with our results, previous research has reported that high LC3, PINK1 and p62 protein levels are associated with triple-negative BC (TNBC) patients and with poor response to chemotherapy, similar results have also been found in colorectal cancer, gastric, malignant melanoma, and esophageal cancer [54,55,56,57,58,59,60,61]. This demonstrates that deficiency of mitochondrial activity correlates with the invasiveness and metastatic capacity of the basal-like subtype of BC [62].

In addition, researchers have found that FUNDC1, a mammalian mitophagy receptor, regulates mitochondrial fusion-fission and mitophagy through the interaction of DNM1L/Drp1 and Opa1 and can also recruit LC3 to induce mitophagy [63]. However, it was not identified in this work, but the FUN14 domain containing 2 (FUNDC2) protein was identified, which is also associated with mitochondria autophagy [64]. In a previous study, FUNDC2 gene overexpression was found in BC brain metastases [65]; unfortunately, so far, there are few studies on this protein in cancer.

Cancer cells have particular metabolic demands for proliferation and survival. Although the role of mitochondrial dynamics in the regulation of metabolism is still not well elucidated, it has been shown that mitochondrial fusion can promote OXPHOS capacity; thus, mitochondrial fission may be primarily related to a glycolytic phenotype [9]. Metabolic plasticity has been shown in BC to have the ability to promote late-stage tumor cell survival and relapsing tumor and metastasis formation in patients. To investigate the metabolic preference in breast cancer cell lines, we evaluated the expression of AMPK and HIF1α, which have been considered key modulators of oxidative and glycolytic metabolism, respectively [35,66,67,68]. We confirmed that both MCF7 and MDA-MB-231 cell lines exhibit a switch from mitochondrial OXPHOS to glycolytic metabolism (Figure 5D). This preference for glycolytic metabolism in both cell lines of BC may be due to a decrease in the mitochondrial respiratory capacity, which was more evident in basal-like cell line by a marked decrease in mitochondrial respiration and decoupling of oxidative phosphorylation compared to luminal A cell line (Figure 6B,C). Concerning these mitochondrial alterations, here, through proteomic analysis, we showed a unique OXPHOS signature capturing the differential expression of complexes proteins between luminal A and basal-like tumors (Figure 10C), which could be of great use in exploring new therapeutic targets for BC [69]. This signature demonstrates a lower expression mainly of complexes I and V in basal-like tumors than luminal A tumors. Our results agree with a previous study by Lunetti et al. [70] in which it was suggested that complex I deficiency appears to be compensated by increased complex IV activity [70]. Supporting the idea of the therapeutic application of OXPHOS targeted inhibitors, our data show concordance with this approach, which could also help to sensitize advanced BC. For instance, Raninga et al. [71] demonstrated that marizomib (Mzb), a proteasome inhibitor, inhibits complex II, which leads to reduced OXPHOS, induces caspase-3 dependent apoptosis and reduce tumor growth in TNBC, and reduces metastases in lung and brain cancers [71]. Another approach is the use of metformin as an anticancer therapy, showing that metformin reduces mitochondrial metabolite levels and inhibits complex I of the mitochondrial respiratory chain. However, the mechanism of action of metformin is still unclear; studies suggest that it may activate AMPK o reduce phosphorylated protein kinase B (pAKT) [42,72].

It is well established that alterations in the respiratory chain and the increase in HIF1α, demonstrated here, can increase ROS production, and might be involved as initiators, promoters, and neoplastic transformation since it can act as mutation-driving agents and interact with signaling pathways [73,74]. It has also been suggested that increased ROS generation may play an important role in autophagy by activating signaling pathways [75]. We demonstrated that basal-like cell line produces more significant ROS levels than luminal A cell line (Figure 7A,B). This increase in ROS production may be associated with the impairment of mitochondrial complex I [70]. These differences in ROS production between the subtypes we found correlate with previous studies demonstrating that increased ROS production in TNBC cell lines has a protumorigenic role in the oncogenic signaling necessary for their proliferation and survival and maybe to promote metastatic potential [37,70].

On the other hand, there are drawbacks such as genetic and phenotypic drift and the lack of ability to reflect the intertumoral heterogeneity that cell lines present [76,77]. In this work, we demonstrate that exploiting proteomic data from repositories can be a valuable strategy/alternative that allowed us to integrate and deepen into the process of mitochondrial dynamics and bioenergetics. This strategy allowed us to obtain mitochondrial proteome expression profiles that distinguish between luminal A and basal-like tumors and discriminate between disease and normality (Figure 9). Overall, analysis of the mitochondrial proteome shows us that: (1) mitochondria play an essential role in the development of BC; (2) mitochondrial alterations are associated with increased aggressiveness and metastatic potential of this disease; (3) mitochondria may reflect functional differences between luminal A and basal-like subtypes; and (4) mitochondria proteome may be complementary to the molecular classification of BC, and of the great utility for patient stratification and the search for the new markers of diagnostic and therapeutic value. We believe that the inclusion of mitochondrial biomarkers in BC molecular signatures, such as PAM50, Oncotype DX, MammaPrint, and IHC4 [78], is crucial to improving the capture of intrinsic differences between molecular subtypes, which would have an impact on the diagnosis and even treatment of patients. This is because these signatures are still not sufficient to distinguish between subtypes, as shown in Figure S1A, where it can be seen that some luminal A are mistaken with basal-like tumors.

Therefore, in this work, we propose two mitochondrial signatures that allow us to distinguish between luminal A and basal type breast cancer tumors, from which potential biomarkers for diagnostic and/or therapeutic use could be identified.

## 5. Conclusions

Despite limitations of using cell lines, here it is demonstrated that through an appropriate selection, it was possible to reflect the differences between the luminal A and basal-like and the high heterogeneous that characterize BC. In addition, we demonstrated that through the complementarity of functional studies in BC cell lines and exploiting Mertins et al. [10] proteomics data analyses of BC samples, a functional mitochondrial proteomic signature was generated to distinguish between luminal A and basal-like subtypes of BC.

Furthermore, we show that the imbalance of mitochondrial dynamics may be associated with the loss of mitochondrial quality control and with alterations in bioenergetics in luminal A and basal-like subtypes of BC. We show that although both breast cancer lines representing luminal A and basal-like subtypes possess a Warburg phenotype, the MCF7 (luminal A) cell line was characterized by a more conserved bioenergetics efficiency, even with the capacity to generate ATP via OXPHOS. In contrast, the MDA-MB-231 (basal-like) cell line showed a significant decrease in mitochondrial respiration and a marked uncoupling of OXPHOS. It was also shown that both cell lines were characterized by a significant increase in ROS, which may be due to alterations in OXPHOS complexes, mainly in CIII, CIV and CV.

## Data Availability

Global proteomics data from women with breast cancer classified according to the molecular classification of breast cancer can be found in the publication “Proteogenomics connects somatic mutations to signaling in breast cancer” by Mertins et al. in Table S3 of the supplementary material. Data used in this publication were generated by the Clinical Proteomic Tumor Analysis Consortium (NCI/NIH).

## Supplementary Materials

The following supporting information can be downloaded at: https://www.mdpi.com/article/10.3390/biom12030379/s1, Figure S1: Flow chart of the statistical and bioinformatic analysis process of proteomic data of breast cancer tumors, Figure S2: Hierarchical cluster analysis, Table S1: Antibodies for Western blot and reagents, Table S2: Mitochondrial proteins, Table S3: Mitochondrial proteins expressed in Luminal A and basal-like subtypes of breast cancer, Table S4: Abundance data for PCA, Table S5: Explained variance of PCA, Table S6: Correlation matrix, Table S7: Proteins for analysis of overrepresentation biological processes, Table S8: Overrepresentation of biological processes.
