# Supplementary material for: Characterization of Mitochondrial Proteome and Function in Luminal A and Basal-like Breast Cancer Subtypes Reveals Alteration in Mitochondrial Dynamics and Bioenergetics Relevant to Their Diagnosis
Source: Biomolecules. 2022 Feb 28;12(3):379. doi: 10.3390/biom12030379 (PMC8945677; doi:10.3390/biom12030379)
Supplement: Supplementary file 1 [file biomolecules-12-00379-s001.zip › biomolecules-1565316-supplementary/Ariadna et al 2022_Supplementary_Figures.pdf]

Supplementary figures to

Characterization of mitochondrial proteome and function in luminal A and basal-like breast cancer subtypes reveals alteration in mitochondrial dynamics and bioenergetics relevant to their diagnosis

Ariadna Jazmín Ortega-Lozano [1], Leopoldo Gómez-Caudillo [1], Alfredo Briones-Herrera [1], Omar Emiliano Aparicio-Trejo [2] and José Pedraza-Chaverri [1,*]

[1] Department of Biology, Faculty of Chemistry, National Autonomous University of Mexico (UNAM), Mexico City 04510, Mexico; arijol-sk@hotmail.com (A.J.O.-L); pologoca@gmail.com (L.G.-C); bhalfredo@gmail.com (A.B.H).

[2] Department of Cardio-Renal Physiopathology, National Institute of Cardiology "Ignacio Chávez", Mexico City 14080, Mexico; emilianoaparicio91@gmail.com (O.E.A.-T.).

* Correspondence: pedraza@unam.mx; Tel./Fax: +52-55-5622-3878

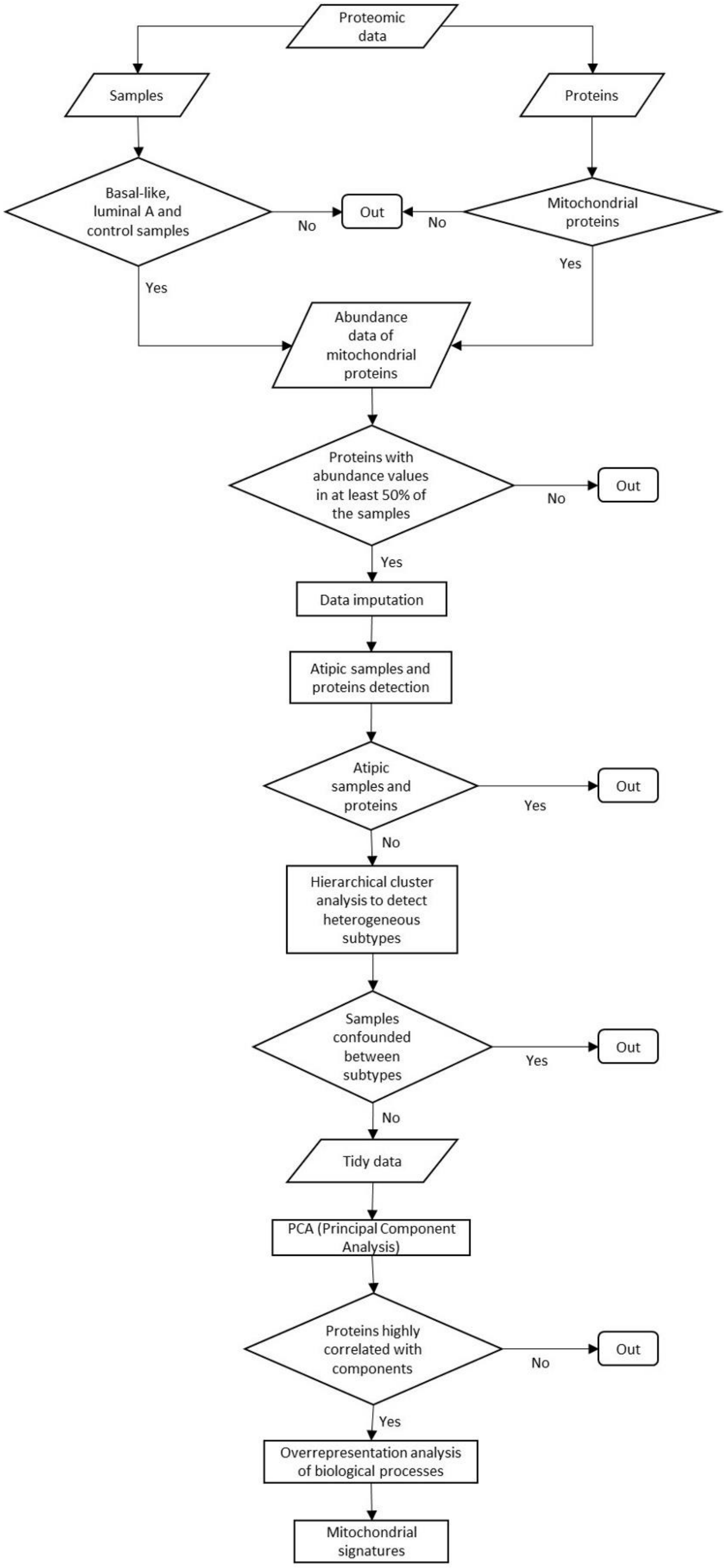

Figure S1. Flow chart of the statistical and bioinformatic analysis process of proteomic data of breast cancer tumors.

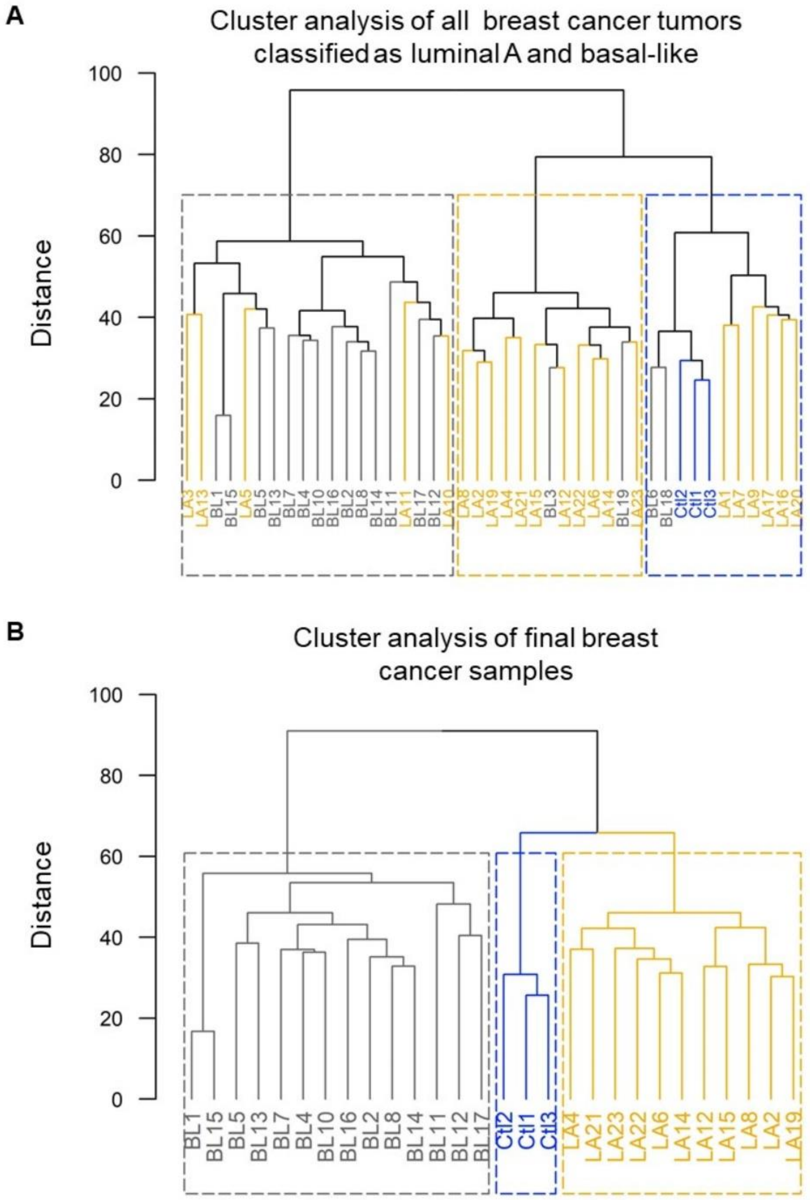

Figure S2. Hierarchical cluster analysis. (A) Hierarchical analysis with 3 control samples (Ctl), 22 luminal A (LA) breast cancer samples and 18 basal-likes (BL) breast cancer samples, in which it is observed that despite the molecular classification of breast cancer patients, there are 10 luminal A samples and 4 basal-like samples that confuse with other molecular subtypes. (B) Elimination of the 14 breast cancer tumors, obtaining homogeneous groups for the luminal A and basal-like subtypes of breast cancer.
